# Molecular Characterization of Velogenic Newcastle Disease Virus (Sub-Genotype VII.1.1) from Wild Birds, with Assessment of Its Pathogenicity in Susceptible Chickens

**DOI:** 10.3390/ani11020505

**Published:** 2021-02-15

**Authors:** Khaled Saad Abd Elfatah, Moshira Abas Elabasy, Faris El-khyate, Ehab Kotb Elmahallawy, Samah M. Mosad, Fatma A. El-Gohary, Walied Abdo, Ashraf Al-Brakati, Mohamed G. Seadawy, Abd Elnaby Tahoon, Abd Elgalil El-Gohary

**Affiliations:** 1 Department of Poultry and Rabbit Diseases, Faculty of Veterinary Medicine, Kafrelsheikh University, Kafrelsheikh 33511, Egypt; khaledelagamy1985@yahoo.com (K.S.A.E.); moshera.elabassi@vet.kfs.edu.eg (M.A.E.); fareselkhayat@yahoo.com (F.E.-k.); abdelgalil.elgouhari@vet.kfs.edu.eg (A.E.E.-G.); 2 Department of Zoonoses, Faculty of Veterinary Medicine, Sohag University, Sohag 82524, Egypt; 3 Department of Virology, Faculty of Veterinary Medicine, Mansoura University, Mansoura 35516, Egypt; dr.sama786@yahoo.com or; 4 Department of Hygiene and Zoonoses, Faculty of Veterinary Medicine, Mansoura University, Mansoura 35516, Egypt; dr.fatmagohary@gmail.com; 5 Department of Pathology, Faculty of Veterinary Medicine, Kafrelsheikh University, Kafrelsheikh 33511, Egypt; waliedsobhy@yahoo.com; 6 Department of Human Anatomy, College of Medicine, Taif University, P.O. Box 11099, Taif 21944, Saudi Arabia; a.albrakati@tu.edu.sa; 7 Main Chemical Laboratories, Egyptian Army, Cairo 11351, Egypt; Biologist202054@yahoo.com; 8 Animal Health Research Institute, Kafrelsheikh 33511, Egypt; tahoon63@yahoo.com

**Keywords:** wild birds, NDV sub-genotype VII.1.1, RT-PCR, phylogenetic analysis, Egypt

## Abstract

**Simple Summary:**

Newcastle disease virus (NDV) is a highly contagious viral disease affecting a wide range of avian species. The disease can be particularly virulent in chickens, resulting in high mortality and morbidity. In this study, we characterized velogenic NDV sub-genotype VII.1.1 from wild birds and assessed its pathogenicity in susceptible chickens. One hundred wild birds from the vicinity of poultry farms with a history of NDV infection were examined clinically. Pooled samples from the spleen, lung, and brain were screened using real-time reverse transcriptase polymerase chain reaction (RRT-PCR) and reverse transcriptase polymerase chain reaction (RT-PCR) to detect the NDV F gene fragment, and phylogenetic analysis was carried out for identification of the genetic relatedness of the virus. Chickens were infected with the strains identified, and the major histopathological changes were assessed. Interestingly, NDV was detected in 44% of cattle egret samples and 26% of house sparrow samples by RRT-PCR, while RT-PCR detected NDV in 36% of cattle egrets examined and 20% of house sparrow samples. Phylogenetic analysis revealed close identity, of 99.7–98.5% (0.3–1.5% pairwise distance), between the isolates used in our study and other Egyptian class II, sub-genotype VII.1.1 NDV strains. Histopathological examination identified marked histopathological changes that are consistent with NDV. These findings provide interesting data in relation to the detection of NDV sub-genotype VII.1.1 in wild birds and reveal the major advantages of the combined use of molecular and histopathological methods in the detection and characterization of the virus. More research is needed to determine the characteristics of this contagious disease in the Egyptian environment.

**Abstract:**

Newcastle disease (ND) is considered to be one of the most economically significant avian viral diseases. It has a worldwide distribution and a continuous diversity of genotypes. Despite its limited zoonotic potential, Newcastle disease virus (NDV) outbreaks in Egypt occur frequently and result in serious economic losses in the poultry industry. In this study, we investigated and characterized NDV in wild cattle egrets and house sparrows. Fifty cattle egrets and fifty house sparrows were collected from the vicinity of chicken farms in Kafrelsheikh Governorate, Egypt, which has a history of NDV infection. Lung, spleen, and brain tissue samples were pooled from each bird and screened for NDV by real-time reverse transcriptase polymerase chain reaction (RRT-PCR) and reverse transcriptase polymerase chain reaction (RT-PCR) to amplify the 370 bp NDV F gene fragment. NDV was detected by RRT-PCR in 22 of 50 (44%) cattle egrets and 13 of 50 (26%) house sparrows, while the conventional RT-PCR detected NDV in 18 of 50 (36%) cattle egrets and 10 of 50 (20%) of house sparrows. Phylogenic analysis revealed that the NDV strains identified in the present study are closely related to other Egyptian class II, sub-genotype VII.1.1 NDV strains from GenBank, having 99.7–98.5% identity. The pathogenicity of the wild-bird-origin NDV sub-genotype VII.1.1 NDV strains were assessed by experimental inoculation of identified strains (KFS-Motobas-2, KFS-Elhamoul-1, and KFS-Elhamoul-3) in 28-day-old specific-pathogen-free (SPF) Cobb chickens. The clinical signs and post-mortem changes of velogenic NDV genotype VII (GVII) were observed in inoculated chickens 3 to 7 days post-inoculation, with 67.5–70% mortality rates. NDV was detected in all NDV-inoculated chickens by RRT-PCR and RT-PCR at 3, 7, and 10 days post-inoculation. The histopathological findings of the experimentally infected chickens showed marked pulmonary congestion and pneumonia associated with complete bronchial stenosis. The spleen showed histocytic cell proliferation with marked lymphoid depletion, while the brain had malacia and diffuse gliosis. These findings provide interesting data about the characterization of NDV in wild birds from Egypt and add to our understanding of their possible role in the transmission dynamics of the disease in Egypt. Further research is needed to explore the role of other species of wild birds in the epidemiology of this disease and to compare the strains circulating in wild birds with those found in poultry.

## 1. Introduction

Newcastle disease (ND) is a highly contagious disease caused by Newcastle disease virus (NDV) [1]. This disease is ranked the third-most-significant poultry disease, having been reported in 109 member countries of the World Organization for Animal Health (OIE) [2,3]. The disease has attracted the attention of several researchers over the past decades due to its global impact on the poultry industry [1,2,3,4]. The frequent incidence of NDV infection, even in vaccinated birds, is due to improper vaccination and may also be associated with mutations of the virus that alter its biological properties and virulence [5,6,7]. According to the OIE, the most virulent strains of the virus are fatal and their intracerebral pathogenicity index is around 0.7 or higher [8].

Avian paramyxoviruses 1 and ND viruses were classified by the International Committee on Taxonomy of Viruses as Avian orthoavula virus serotype 1 (formerly Avian avulavirus 1) in the new subfamily Avulavirinae and family Paramyxoviridae [9]. To the best of the author’s knowledge, three strains of the NDV are known: lentogenic, mesogenic, and velogenic. Among these, the velogenic strain is considered to be the most virulent, producing high mortality and severe respiratory and nervous symptoms [10]. NDV is divided into class I NDV strains, grouped into a single genotype and 3 sub-genotypes, and class II NDV strains, divided into at least 20 distinct genotypes (I–XXI) made up of several sub-genotypes [11]. Genotype VII is subdivided into three sub-genotypes [12], while genotypes I, V, VI, VII, XII, XIII, XIV, and XVIII are each divided into several sub-genotypes [11,13,14,15]. The fourth NDV panzootic was caused by viruses of a single genotype (VII.1.1) that includes former sub-genotypes VIIb, VIId, VIIe, VIIj, and VIIl; sub-genotype VIIf is considered to be a separate sub-genotype, VII.1.2 [11]. The groups of viruses involved in the fifth NDV panzootic that affected Africa, Asia, the Middle East, and Europe [16,17,18,19,20,21], were merged into a single sub-genotype, VII.2, together with five other sequences identified as sub-genotype VIIk and then also assigned to VII.2 [22]. The predominant NDV sub-genotype in Egypt is VIId (VII.1.1), which has led to several outbreaks in poultry [23]. The disease is a significant biosecurity risk in NDV-free zones, where sporadic outbreaks might have significant impacts on trade. NDV remains one of the major causes of huge economic losses, is a harmful pathogen for poultry breeding, and possesses limited zoonotic importance [24].

ND is highly contagious, affecting more than 250 domestic and wild bird species, as well as reptiles and humans [1,25,26]. The infection is transmitted through exposure to fecal matter and other excretions of infected birds, as well as direct and indirect contact with contaminated food, water, and utensils [27]. The main reservoirs of virulent strains are poultry, while wild birds, such as house sparrows, crows, hawks, and waterfowls, could harbor the low-virulent strains [28,29,30]. However, virus exchange among wild birds produces high risk for both bird populations [30,31], as some viruses pose threats when introduced into new geographic locations and new host species [30,31,32].

As mentioned above, a wide range of wild bird species can contract the infection by NDV strains with varying degrees of pathogenicity and genetic diversity [21,24,33]. Cattle egrets are susceptible to infection with velogenic viscerotropic NDV (VVNDV) and act as potential carriers in the transmission of VVNDV among poultry flocks [34]. However, the vast majority of the NDV genotypes reported in wild birds rarely result in severe or clinically significant lesions in infected birds [35]. Despite this fact, understanding the extent of the viral burden and the pathotypic and genotypic characteristics is valuable for assessing the possible risks of emerging disease and consequently developing appropriate control measures for combating the disease [36].

The present study was initially undertaken to assess the molecular nature of the NDV genotype circulating in cattle egrets and house sparrows associated with recent outbreaks in poultry flocks in Kafrelsheikh Governorate, Egypt.

## 2. Material and Methods

### 2.1. Ethical Considerations

Ethical approval was obtained from the Research, Publication and Ethics Committee of the Faculty of Veterinary Medicine, Kafrelsheikh University, Egypt. The research complied with all relevant Egyptian legislation. The ethical approval number is KFS 2017/3. Research and all experimental procedures were performed in accordance with the pertinent guidelines concerning animal handling, following international and national guidelines for animal care and welfare.

### 2.2. Study Area, Sample Collection, and Sample Preparation

The study was conducted from October 2017 to October 2019 (Table 1). Fifty cattle egrets and fifty house sparrows (total *N* = 100) were sampled from the vicinity of poultry farms with a history of NDV infection in El-Hamoul, Kafrelsheikh, Balteem, and Motobas cities, Kafrelsheikh Governorate, Egypt. The birds were trapped alive overnight, using nets, from the trees around poultry farms suspected to be infected with velogenic NDV. The wild birds were euthanized and slaughtered humanely after intravenous injection of diazepam tranquilizer (2.5 mg/kg) to reduce stress, as previously described [37]. Birds were clinically examined, and clinical signs and post-mortem changes were recorded. Lung, spleen, and brain tissue samples were aseptically collected and pooled from each bird. Tissue samples from the lung, spleen, and brain were also pooled from three healthy chickens, confirmed to be NDV-free by RT-PCR, as negative control samples. Samples from the wild birds and negative control samples were homogenized in phosphate-buffered saline (pH 7.2) with an antibiotic mixture (50 IU/mL penicillin and 50 µg/mL streptomycin) and mycostatin as an antifungal (50 mg/mL). Tissue homogenates were then centrifuged at 2000 rpm for 10 min, and the clarified supernatants were collected and stored at −80 °C until further use in virus isolation and viral RNA extraction [38]. The wild birds were confirmed using real-time reverse transcriptase polymerase chain reaction (RRT-PCR) to be free from other infectious agents causing diarrhea, such as avian influenza, infectious bursal disease virus, and salmonellosis.

### 2.3. Standard NDV

The standard velogenic mans1 NDV strain (GenBank accession no. MN537832) from a previous study was used as a positive control sample in RRT-PCR and RT-PCR [39]. This velogenic mans1 NDV strain was a field strain isolated from 45-day-old broiler chickens from the Dakahalia Governorate, Egypt [39].

### 2.4. Molecular Characterization of NDV

#### 2.4.1. Viral RNA Extraction

Viral RNA was extracted from the supernatant fluids of homogenized pooled lung, spleen, and brain samples collected from cattle egrets and house sparrows and positive and negative control samples using commercial kits (QIAamp^®^ MinElut^®^ Virus Spin Kit; QIAGEN GmbH, Hilden, Germany) in accordance with the manufacturer’s guidelines. The extracted RNA was then stored at −80 °C until further use.

#### 2.4.2. Real-Time Reverse Transcriptase Polymerase Chain Reaction

QuantiTect Probe RT-PCR Master Mix (QIAGEN, Qiagen Str. 1, 40,724 Hilden, Germany) was used for RRT-PCR amplifications of the NDV F gene fragment (101 bp) from extracted RNA in accordance with kit guidelines. RRT-PCR amplification of velogenic and mesogenic strains of the NDV F gene fragment was conducted using a previously reported set of primers, as shown in Table 2 [40]. RT-PCR amplification was performed in a final volume of 25 µL, containing 7 µL of RNA template, 12.5 µL of 2× QuantiTect Probe RT-PCR Master Mix, 3.625 µL of PCR-grade water, 0.25 µL (50 pmol conc.) of each primer (F+4839 and F-4939), 0.125 µL (30 pmol conc.) of the probe (F+4894 (VFP-1)), and 0.25 µL of QuantiTect RT Mix. A Stratagene MX3005P real-time PCR machine was adjusted to 50 °C for 30 min (reverse transcription) and then 94 °C for 15 min (primary denaturation), followed by 40 cycles of denaturation at 94 °C for 15 s, annealing at 52 °C for 30 s, and extension at 72 °C for 10 s.

#### 2.4.3. Reverse-Transcriptase Polymerase Chain Reaction

**Figure 1:**
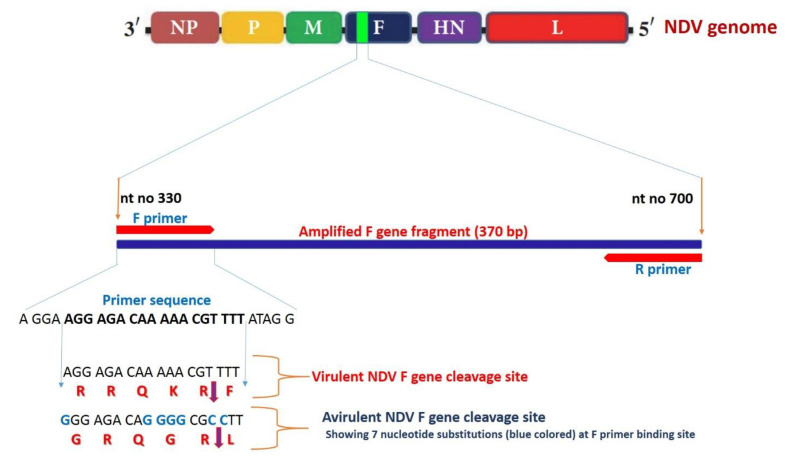
The diagram illustrates RT-PCR targeting 370 bp F gene fragment (nt no. 330–700) with F primer containing a characteristic virulent Newcastle disease virus (NDV) F gene cleavage site (nt no. 334–351). The low-virulent NDV cleavage site showed seven nucleotide substitutions (guanine instead of adenine at positions 334, 342, 343, 344, and 345 and cytosine instead of thiamine at positions 348 and 349) when compared to the forward primer nucleotide sequence, making it difficult to amplify the target fragment from low-virulent NDV.

QIAGEN OneStep RT-PCR Kits (QIAGEN, Qiagen Str. 1, 40,724 Hilden, Germany) were used for RT-PCR amplification of the virulent NDV F gene fragment (400 bp) from the extracted RNA in accordance with kit guidelines. RT-PCR amplification of the virulent NDV F gene fragment was conducted using a previously reported set of primers (Figure 1 and Table 2) [41]. The 50 µL reaction mixture consisted of 10.0 µL of 5x QIAGEN OneStep RT-PCR Buffer, 1 µL (10 pmol) of each primer (NDV-F330 and NDV-R700), 2 µL of deoxyribonucleotides tri-phosphate (dNTPs) mix (10 mM of each dNTP), 2 µL of QIAGEN OneStep RT-PCR Enzyme Mix, 5 μL of extracted RNA, and RNase-free water up to 50 µL. The PCR protocol was performed on a T3 Biometra thermal cycler (Germany) as follows: a single cycle of initial denaturation at 94 °C for 2 min, followed by 40 cycles of denaturation at 95 °C for 30 s, and annealing at 50 °C for 45 s, and the reaction was completed by a final extension at 72 °C for 1 min, with a final incubation step at 72 °C for 10 min. After amplification, 5 µL of PCR products were analyzed by gel electrophoresis (100 volts for 40 min) in 1.5% agarose gel in 0.5 X Tris-Borate Ethylenediaminetetraacetic acid (EDTA) buffer with 0.5 µg/mL of ethidium bromide, against a 100 bp DNA ladder (Jena Bioscience, Germany), after which the DNA bands were visualized with a UV transilluminator.

#### 2.4.4. Sequencing and Phylogenetic Analysis of the Selected Samples

The RT-PCR products of three selected samples (sharp bands) were excised from the gel, and their DNA was purified with QIAquick PCR gel purification kits (QIAGEN, Valencia, CA, USA) in accordance with the manufacturer’s guidelines. The purified DNA from PCR products of the selected samples was sequenced using the Sanger method, using Seqscape^®^ software for raw data analysis. The nucleotide sequences were then placed in GenBank (http://www.ncbi.nlm.nih.gov/Genbank accessed on 12 December 2020) with accession numbers MT878465 (KFS-Elhamoul-1 strain), MT878466 (KFS-Motobas-2 strain), and MT878467 (KFS-Elhamoul-3 strain), as shown in Table 3. ClustalW2 (https://www.ebi.ac.uk/Tools/msa/clustalw2/ accessed on 12 December 2020) was used for the analysis of the sequences. The output alignment files were used for phylogenic maximum-likelihood analysis, with 1000 repeat bootstrap tests in MEGA X software [42]. The obtained nucleotide and deduced amino acid sequences were aligned with other sequences from GenBank using the Clustal W algorithm of BioEdit software Version 7.1, with the Damietta6 strain as a reference strain [43].

### 2.5. Pathogenicity Testing of Wild Bird Sub-Genotype VII.1.1 NDV Strains in Susceptible Chickens

#### 2.5.1. Susceptible Chickens

One-day-old commercial Cobb 500^®^ chicks (*n* = 160) were purchased from a certified local commercial hatchery and housed in separate pens with all appropriate biosecurity restrictions. Pens were physically separated from each other to avoid transmission of the infection between groups. Water and food were provided ad libitum throughout the experimental period. The chicks were kept for 28 days without any medication or vaccination. All chicks were bred according to the experimental animal care and welfare guidelines of the Animal Health Research Institute, Kafrelsheikh, Egypt. The 28-day-old chickens were used for assessment of the pathogenicity of the strains with genotype VII.1.1 originating in wild birds.

#### 2.5.2. NDV Strains

Three sub-genotype VII.1.1 NDV strains were identified, two strains from cattle egrets (KFS-Motobas-2 and KFS-Elhamoul-1) and one strain from house sparrows (KFS-Elhamoul-3), and were used in experimental infection of susceptible chickens.

#### 2.5.3. Virus Propagation in Embryonated Chicken Eggs

Specific-pathogen-free 10-day-old embryonated chicken eggs were obtained from an Egyptian SPF egg production farm (Nile SPF), Fayoum, Egypt. About 0.2 mL of supernatant fluid from each sample (KFS-Motobas-2, KFS-Elhamoul-1, and KFS-Elhamoul-3 strains) and a negative control sample from normal healthy chickens were inoculated into the allantoic cavity of the SPF-ECEs (five ECEs per sample) for three successive passages. Inoculated eggs were incubated at 37 °C for 5 days; dead embryos at 24 h post-inoculation (PI) were eliminated. Eggs with dead embryos 2–5 days PI were collected and examined for embryonic lesions, and their allantoic fluids were collected. Live embryos 5 days PI were preserved at 4 °C for 12 h, and then the allantoic fluids from the dead embryos 2 to 6 days PI were collected and used for further egg passage. The allantoic fluids collected from the third egg passage were preserved at −80 °C until use in virus titration and experimental infection of susceptible chickens [44].

#### 2.5.4. Virus Titration

Three sub-genotype VII.1.1 NDV strains identified in the present study were titrated in SPF-ECEs. Tenfold serial dilutions were prepared from each strain using allantoic fluids collected from the third egg passage, and 0.2 mL from each dilution was inoculated into the allantoic cavity of 10-day-old ECEs (five ECEs per sample). Five days PI, embryo infective dose 50 (EID50) was calculated, as previously described [45]. The virus titers were 10^7^, 10^8.6^, and 10^7.3^ EID50/mL for the KFS-Motobas-2, KFS-Elhamoul-1, and KFS-Elhamoul-3 strains, respectively.

#### 2.5.5. Experimental Design

Chickens used for experimental design were divided into four groups (G1–G4) of 40 birds each at 28 days old. Each group was kept in a separate pen with strict biosecurity measures to avoid cross infection. Taking into account that intramuscular inoculation does not mimic the natural route of exposure, groups G1–G3 were experimentally infected with KFS-Motobas-2, KFS-Elhamoul-1, and KFS-Elhamoul-3 sub-genotype VII.1.1 NDV strains, respectively, by intramuscular inoculation (dose = 10^6^ EID50/mL). Birds in G4 were inoculated intramuscularly with negative control samples of allantoic fluid from the third egg passage [8,46]. Infected chickens were observed daily for 10 days post-infection (dpi), and mortality, clinical signs, and pathological lesions were recorded. At 3, 7, and 10 dpi, three birds were randomly selected from each group and euthanized, and lung, spleen, and brain tissue samples were aseptically collected for NDV detection using RRT-PCR and RT-PCR, as previously described [40,41]. At the fifth dpi, lung, spleen, and brain tissue samples were aseptically collected from freshly dead and/or euthanized birds (three birds per group) and fixed in 10% neutral formalin for histopathological examination.

#### 2.5.6. Histopathological Examination

Formalin-fixed lung, spleen, and brain tissue samples from experimentally infected chickens were dehydrated, and embedded in paraffin wax. Tissue sections (5 μm) were then de-paraffinized, stained with hematoxylin and eosin stain, and microscopically examined [47].

## 3. Results

### 3.1. Clinical Signs and Post-Mortem Lesions

In the present study, most of the clinically examined cattle egrets and house sparrows were apparently healthy with no clinical signs. However, four cattle egrets and three house sparrows showed ruffled feathers, with three cattle egrets and two house sparrows also showing whitish-green diarrhea. The post-mortem examination of euthanized birds showed enteritis, whitish-green intestinal contents, and cloudiness of the air sacs in a few birds.

### 3.2. Molecular Characterization of NDV

#### 3.2.1. Real-Time Reverse Transcriptase Polymerase Chain Reaction

Of the 50 cattle egrets and 50 house sparrows subjected to RRT-PCR, 22 cattle egret samples (44%) and 13 house sparrow samples (26%) were positive, with cycle threshold (C*_t_*) values ranging from 15.45 to 39.65 (Table 3).

#### 3.2.2. Reverse Transcriptase Polymerase Chain Reaction

Conventional RT-PCR was used to amplify a 370 bp fragment of the NDV F gene from 18 samples (36%) from cattle egrets, 10 samples (20%) from house sparrows, and a positive control sample. The other 32 samples (64%) from cattle egrets, 40 samples (80%) from house sparrows, and a negative control sample showed no band at 370 bp (Table 3).

#### 3.2.3. Sequencing and Phylogenetic Analysis of NDV F Gene Fragment

**Figure 2:**
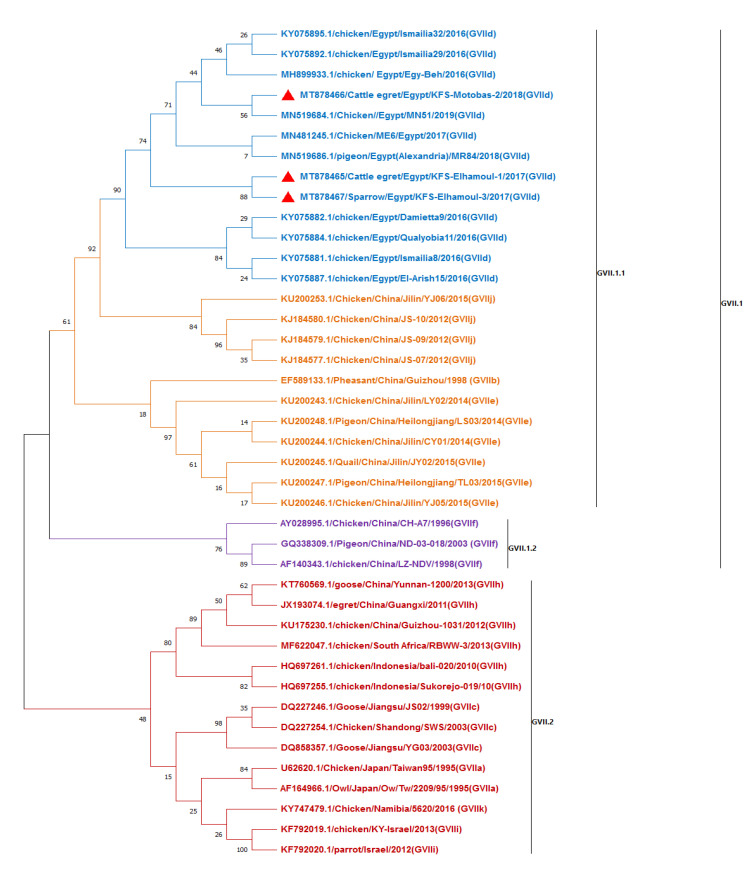
Phylogenetic maximum-likelihood tree of NDV F gene fragment sequences with 1000 bootstrap repeats, including GVII sub-genotypes. Our strains KFS-Motobas-2, KFS-Elhamoul-1, and KFS-Elhamoul-3 (red triangles) were aligned with other GVII.1.1 strains obtained from GenBank.

Three samples (from two cattle egrets and one house sparrow) were selected from the RT-PCR positive samples (sharp bands) for DNA sequencing. The sequences were analyzed in comparison with reference class II GVII sub-genotypes (GVII.1 and GVII.2) of NDV F gene sequences from GenBank (Figure 2). The three strains identified in the present study (KFS-Motobas-2, KFS-Elhamoul-1, and KFS-Elhamoul-3) clustered with other Egyptian strains in sub-genotype VII.1.1 (formerly GVIId). Our strains were closely related to the Egyptian NDV sub-genotype VII.1.1 strains isolated from chickens from different localities in Egypt, including the Dakahlia28, Damietta9, Qualyobia11, El-Arish15, and Ismailia8 strains. The strains KFS-Elhamoul-1 and KFS-Elhamoul-3 were identical (100% identity) and showed 99.1% identity with the KFS-Motobas-2 strain. The present strains KFS-Elhamoul-1 and KFS-Elhamoul-3 showed 98.8% identity with the Damietta9, Qualyobia11, and Ismailia8 strains and 98.5% identity with the Dakahlia28 and El-Arish15 strains. The KFS-Motobas-2 strain showed 99.1% identity with the Damietta9, Qualyobia11, and Ismailia8 strains and 98.8% identity with the Dakahlia28 and El-Arish15 strains. Analysis of the GVII sub-genotypes revealed that the GVII sub-genotypes are highly divergent, with 10.7% pairwise distance between the Namibia-5620 (GVII.2) strain and the Egyptian GVII.1.1 strains (KFS-Elhamoul-1, KFS-Elhamoul-3, MN51, and MR84) (Figure 2 and Table S1).

**Figure 3:**
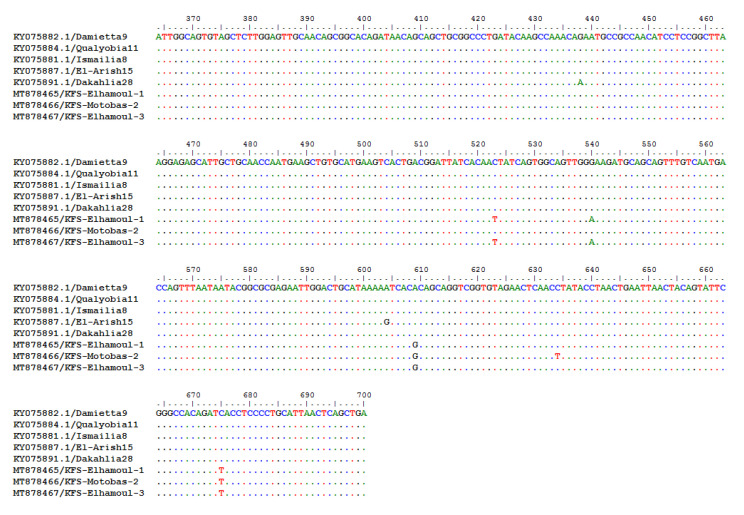
BioEdit Clustal W algorithm alignment of the Egyptian GVII 1.1 strain’s nucleotide sequences showed G609A and T675C nucleotide substitutions in our three identified strains, T523C and A540G nucleotide substitutions in KFS-Elhamoul-1 and KFS-Elhamoul-3 strains, and T634C nucleotide substitution in KFS-Motobas-2.

**Figure 4:**
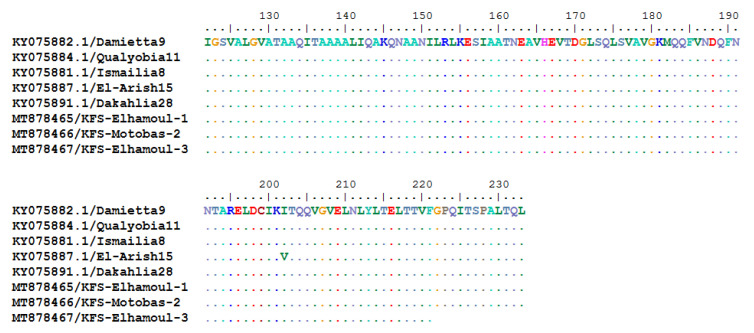
BioEdit Clustal W algorithm alignment of the Egyptian GVII.1.1 strain’s deduced amino acid sequences showing no amino acid substitution in our strains (KFS-Motobas-2, KFS-Elhamoul-1, and KFS-Elhamoul-3).

The BioEdit nucleotide and the deduced amino acid sequences aligned with the Damietta6 strain as a reference strain revealed that the three strains identified in this study had two common nucleotide substitutions (G609A and T675C). The KFS-Elhamoul-1 and KFS-Elhamoul-3 strains showed two nucleotide substitutions (T523C and A540G), while KFS-Motobas-2 had a single nucleotide substitution (T634C). All of these nucleotide substitutions were silent, without any amino acid substitutions (Figure 3 and Figure 4).

### 3.3. Pathogenicity Testing of Wild Birds’ NDV Sub-Genotype VII.1.1 Strains in Susceptible Chickens

#### 3.3.1. Virus Propagation in Embryonated Chicken Eggs

**Figure 5:**
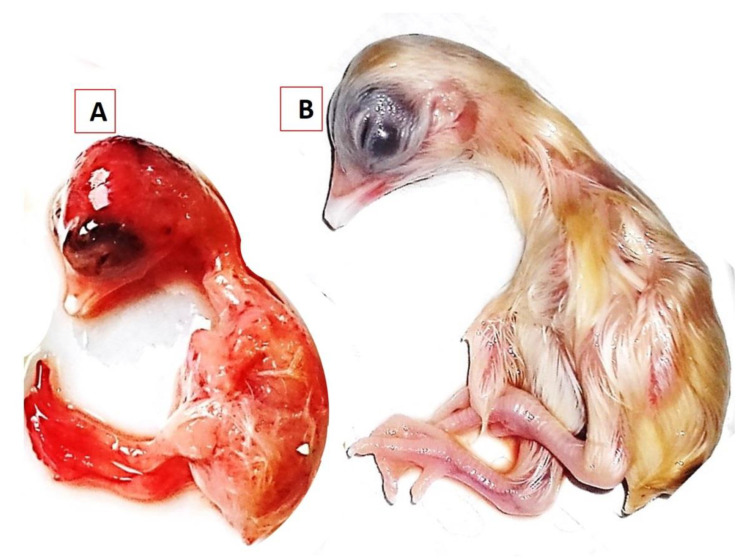
Results of NDV GVII.1.1 isolation in the allantoic cavities of 10-day-oldembryonated chicken eggs. (**A**) NDV sub-genotype VII.1.1 KFS-Motobas-2-strain-inoculated embryo showing dwarfism, abnormal feathering, and congestion, with sub-cutaneous hemorrhages on the head and legs and (**B**) the negative control embryo inoculated with the negative control sample.

KFS-Motobas-2, KFS-Elhamoul-1, and KFS-Elhamoul-3 strains were propagated in 10-day-old ECEs. Inoculated embryos were dwarfed and congested, with sub-cutaneous hemorrhages in the head, legs, and the back area and had edema, abnormal feathering, and gelatinous material on the skin. The embryos’ mortality was recorded at 72 h PI (first and second passages) and 48 h into the third passage (Figure 5).

#### 3.3.2. Clinical Signs and Post-Mortem Changes in Experimentally Infected Chickens

**Figure 6:**
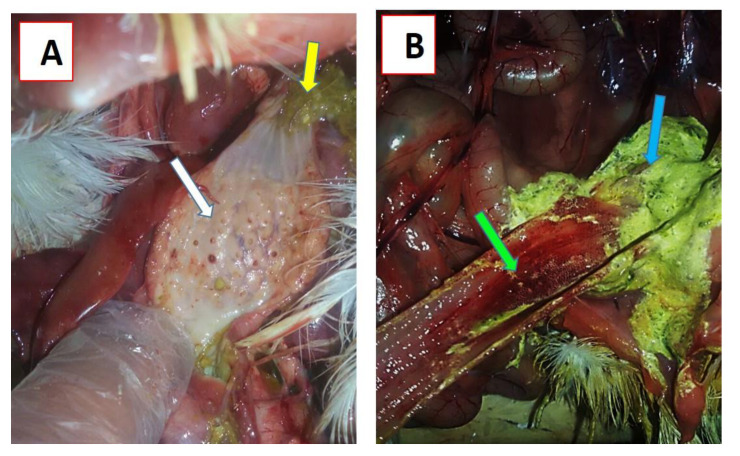
Post-mortem changes in experimentally infected chickens: (**A**) hemorrhages on the tips of proventricular glands (white arrow) and greenish proventricular contents (yellow arrow); (**B**) greenish intestinal contents (blue arrow) and hemorrhagic enteritis (green arrow).

Twenty-eight-day-old Cobb chickens in groups G1 (KFS-Motobas-2), G2 (KFS-Elhamoul-1), and G3 (KFS-Elhamoul-3), together with G4 (negative control group) were clinically examined daily for any clinical signs, mortality, or post-mortem changes in dead birds. Clinical signs started to appear at the third dpi as ruffled feathers, depression, and decrease in feed and water intake, with mortality in some birds. Severe depression was observed during the fourth dpi, with anorexia, greenish diarrhea, a swollen head, and respiratory signs, with a high mortality rate (Table 4). Nervous signs appeared at the fifth dpi, with other, previously mentioned signs. These signs remained until the end of the experiment (tenth dpi). Mortality rates were 80%, 67.5%, and 62.5% in groups G1, G2, and G3, respectively, while group G4 showed no mortality. The post-mortem examination of dead birds showed hemorrhagic tracheitis, lung congestion, enlarged hemorrhagic cecal tonsils, enlarged spleen, hemorrhages on the periventricular gland tips with greenish proventricular contents (Figure 6A), and hemorrhagic intestinal serosal surface with greenish intestinal contents (Figure 6B). NDV was successfully detected by RT-PCR in all tested samples at 3, 7, and 10 dpi from groups G1, G2, and G3, while group G4 was negative (Table 5).

#### 3.3.3. Histopathological Examination

**Figure 7:**
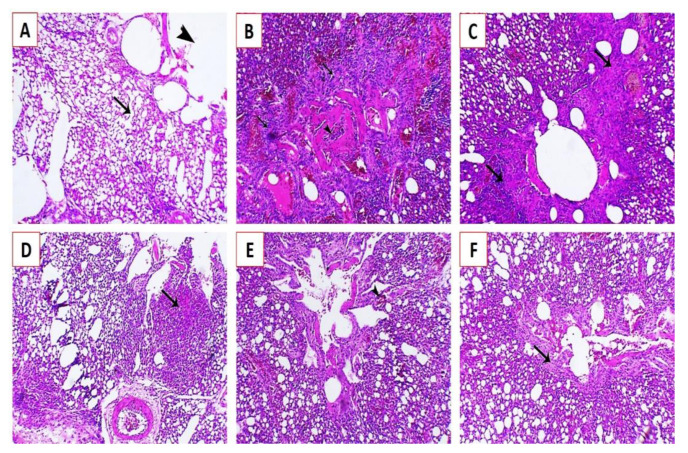
Histopathological lesions in the lungs of experimentally infected Cobb chickens 5 days post-infection (dpi): (**A**) lung of the negative control bird (G4) showing normal parabronchi (arrowhead) and normal air capillaries (arrow); (**B**) lung of an experimentally infected bird in G1 showing congestion of blood capillaries and bronchial obstruction (arrowhead) attributed to peribronchial inflammatory cells infiltration (arrows); (**C**) lung of an experimentally infected bird in G1 showing marked endodermal hyperplasia around the parabronchi (arrows), associated with obvious inflammatory cells infiltration; (**D**) lung of an experimentally infected bird in G2 showing focal pneumonia associated with inflammatory cell infiltration (arrow); (**E**) lung of an experimentally infected bird in G2 showing mild congestion (arrowhead) and mostly patent bronchi and air capillaries; and (**F**) lung of an experimentally infected bird in G3 showing mild endodermal hyperplasia (arrows) and increase in the functional respiratory spaces stained by Hematoxylin and eosin (H&E X200).

Histopathological examination of the lung, spleen, and brain tissue samples collected at the fifth dpi from groups G1, G2, and G3 revealed that the affected lungs showed congestion of blood capillaries and bronchial obstruction attributed to the infiltration of peribronchial inflammatory cells (Figure 7B) with marked endodermal hyperplasia around the parabronchi, associated with obvious inflammatory cell infiltration (Figure 7C). Focal pneumonia associated with infiltration of inflammatory cells was also observed (Figure 7D), with mild congestion and mostly patent bronchi and air capillaries (Figure 7E) and mild endodermal hyperplasia with an increase in the functional respiratory spaces (Figure 7F).

**Figure 8:**
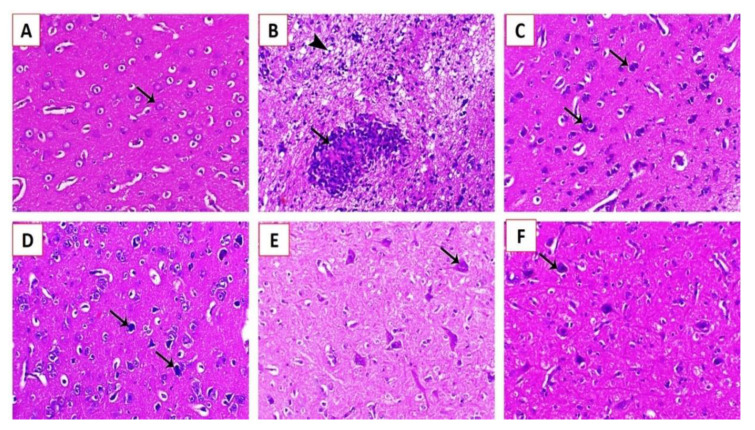
Histopathological lesions in the brains of experimentally infected Cobb chickens 5 dpi: (**A**) brain of the negative control bird (G4) showing normal neuronal cells (arrow); (**B**) brain of an experimentally infected bird in G1 showing spongiosis of the nerve fibers (arrowhead) and focal malacia associated with diffuse and focal conglomerate aggregation of glia cells (arrow); (**C**) brain of an experimentally infected bird in G1 showing gliosis associated with neuronophagia (arrows); (**D**) brain of an experimentally infected bird in G2 showing ischemic neuronal injury (arrows); (**E**) brain of an experimentally infected bird in G3 showing mild to moderate degree of ischemic neuronal injury associated with marked neuronal tigrolysis (arrow); and (**F**) brain of an experimentally infected bird in G3 showing a mild degree of ischemic neuronal injury (arrow) (H&E, X200).

**Figure 9:**
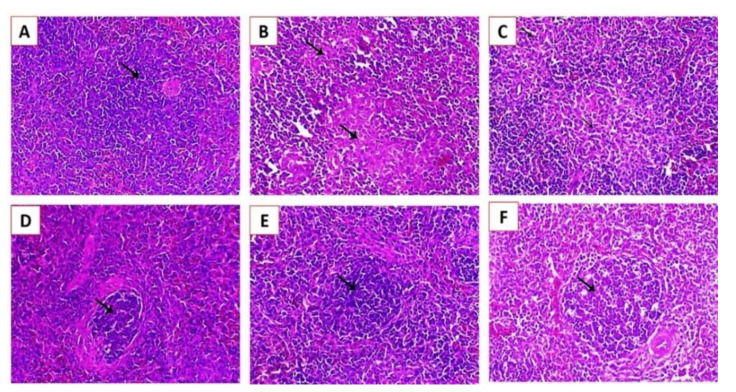
Histopathological lesions in the splenic tissues of experimentally infected Cobb chickens 5 dpi: (**A**) Scheme 4 showing a normal lymphoid follicle with normal lymphocytes around the central arteriole (arrow); (**B**) spleen of an experimentally infected bird in G1 showing marked lymphoid depletion associated with marked histocytic cell proliferation (arrows); (**C**) spleen of an experimentally infected bird in G2 showing marked histocytic cell proliferation (arrows); (**D**) spleen of an experimentally infected bird in G2 showing a normal lymphoid nodule (arrow); (**E**) spleen of an experimentally infected bird in G3 showing an increase in lymphoid cell proliferation within the white pulp (arrow); and (**F**) spleen of an experimentally infected bird in G3 showing increased lymphoid cell proliferation (arrow) (H&E, X200).

The spleen of experimentally infected Cobb chickens 5 dpi showed marked lymphoid depletion associated with marked histocytic cell proliferation in group G1 (Figure 8B) and marked histocytic cell proliferation in group G2 (Figure 8C), and normal lymphoid nodules were also observed (Figure 8D). Moreover, increased lymphoid cell proliferation within the white pulp was also observed (Figure 8E,F). The brain of experimentally infected Cobb chickens 5 dpi showed spongiosis of nerve fibers, with diffuse and focal conglomerate aggregation of glia cells (Figure 9B) and gliosis associated with neuronophagia (Figure 9C). Ischemic neuronal injury was also observed with marked neuronal tigrolysis (Figure 9D–F).

## 4. Discussion

ND is an economically devastating viral disease that affects the poultry industry [5,24]. The disease is considered to be endemic in various areas of the world, such as Central and South America, Asia, the Middle East, and Africa [4]. Wild birds play a critical role in the evolution of NDV [48]. Clearly, surveillance of NDV in wild birds is important to reduce the risk of possible spreading of NDV to poultry flocks.

The present work characterized NDV in wild cattle egrets and house sparrows. The work involved the molecular characterization and phylogenetic analysis of the NDV strains circulating in wild birds collected from Kafrelsheikh Governorate, Egypt. The study also included pathogenicity testing of isolates from chickens, followed by histopathological examination and molecular identification of the identified virus for verification of the results. It is noteworthy to state that several previous reports have documented the role played by different species of wild birds, including cattle egrets (*Bublicus ibis*) and house sparrows (*Passer domesticus*), migratory waterfowl, and other aquatic birds, in the transmission of different strains of NDV [21,28,49,50,51,52,53,54,55,56,57,58,59]. A review of this literature identified the possible release of highly virulent viruses into poultry or wild birds and the existence of epidemiological links between field isolates [20,59,60]. At a national level, a previous study reported and identified two velogenic viscerotropic and one mesogenic pathotype in cattle egrets in the EL-Marg area in Cairo, Egypt [61]. NDV was also characterized in sparrows in several previous reports [49,62,63].

Many efforts have been made over the past decades to develop efficient diagnostic methods, such as virus isolation in embryonated chicken eggs and conventional serological methods using enzyme-linked immunosorbent assay, hemagglutination (HA), and hemagglutination inhibition (HI) tests [5,59,60,62,63,64], but a high incidence of false-positive results and low sensitivity have been reported with these methods [5,64,65,66]. In addition, virus isolates from oropharyngeal or cloacal swabs or tissues from infected birds have been used, but the methods are tedious and time consuming [59,67]. PCR-based assays targeting the amplification of a specific region of the genome of NDV offer many advantages for identification of the virus, besides their important role in differentiation of the tremendous number of virus strains [5,52,68]. Amplification of the NDV F gene using RT-PCR is usually used for NDV detection, and the resulting PCR product can be used for assessment of the virulence of NDV [69]. In the present study, RRT-PCR detected virulent NDV in 22 of 50 cattle egret samples (4%) and 13 of 50 house sparrows (26%), while conventional RT-PCR amplified a 370 bp fragment of the NDV F gene from 18 samples (36%) of cattle egrets and 10 samples (20%) of house sparrows. A previous study in Egypt detected NDV by RT-PCR in 3.6% (4/112) of tested tracheal and cloacal samples [70]. Schelling et al.[71] reported that RT-PCR failed to amplify the NDV RNA extracted from cloacal swabs of 115 different wild bird species.

NDV genotype VII has caused fatal infections in susceptible birds and is thought to be responsible for the fourth major NDV panzootic worldwide [21]. Analysis of GVII sub-types in the present study revealed that the GVII subtypes are highly divergent. We found a 10.7% pairwise distance (89.3% identity) between the Namibia-5620 (GVII.2) strain and the Egyptian GVII.1.1 strains (KFS-Elhamoul-1, KFS-Elhamoul-3, MN51, and MR84). These results were supported by Xue et al. [72], who concluded that NDV genotype VII is the most predominant genotype worldwide, with complex genetic diversity. The three strains identified in the present study (KFS-Motobas-2, KFS-Elhamoul-1, and KFS-Elhamoul-3) were clustered with other Egyptian strains in sub-genotype VII.1.1 (formerly GVIId). The present findings are also consistent with those of Kim et al.[73], who mentioned that NDV genotype VII is the prevalent genotype in the Middle East and that most NDV isolates from wild birds are aligned to this genotype. Another previous study concluded that sub-genotype VII.1.1 is the predominant NDV sub-genotype, causing several outbreaks in Egypt [23]. Dimitrov et al. [11] reported that the viruses responsible for the fourth NDV panzootic were grouped together into a single genotype (VII.1.1). Despite the geographical separation of the hosts and taking into account that our study did not involve analysis of the genetic relatedness of identified strains to that of poultry, this closely related antigenic and genetic analysis of the isolated strains of the NDV may reflect the possible role of wild and migratory birds in maintaining the transmission cycle of the disease [24,28,74].

As mentioned above, NDV is an acute contagious disease affecting birds of all ages [3]. As shown in our work, wild birds were apparently healthy, but a few birds showed ruffled feathers with whitish-green diarrhea, while post-mortem examination showed enteritis, whitish-green intestinal contents, and cloudiness of the air sacs. The present clinical findings are consistent with previous data reporting respiratory, gastrointestinal, circulatory, and nervous signs in infected chickens [75,76]. The clinical signs might vary depending on several factors, such as the pathogenicity of the virus; host factors such as age, species, and immune status; infectious dose duration and extent; and concurrent infections [77].

In the present work, the pathogenicity of sub-genotype VII 1.1 NDV strains from wild birds was assessed through experimental inoculation of identified strains in 28-day-old Cobb chickens. Specific clinical signs and post-mortem changes of velogenic NDV genotype VII were observed in inoculated chickens 3 to 7 days PI, with 62.5–80% mortality rates. NDV was successfully detected by RRT-PCR and RT-PCR in all NDV-inoculated chickens at 3, 7, and 10 dpi. These results are consistent with those of a previous study in Egypt in which NDV was detected in cloacal swabs from chickens challenged with cattle-egret-origin NDV 4–10 dpi [70]. In the same study [70], the authors revealed that the NDV signs that started to appear on the fourth dpi in chickens challenged with cattle-egret-origin NDV were anorexia, depression, mild respiratory sounds, ocular/nasal discharges, and severe neurological disorders, with 100% mortality. Histopathological examination of experimentally infected chickens in the present study revealed that the affected lungs showed severe congestion and mostly patent bronchi and air capillaries. The spleen exhibited increased lymphoid cell proliferation with severe ischemic neuronal injury. Similarly, several previous reports have documented microscopic pictures of chickens challenged with cattle-egret-origin NDV that have revealed severe histopathological changes in lungs, such as congested blood vessels, pneumonia, focal pulmonary hemorrhage, and mononuclear infiltration of the air capillaries [70,78]. The spleen showed marked depletion with fibrinoid and lymphocytic necrosis, while the brain exhibited congested blood vessels, neuronal edema, and necrotic neurons, with neuronophagia consistent with several previous reports [78,79].

## 5. Conclusions

The present study reinforced the importance of the combined use of molecular methods and pathogenicity testing for the characterization and identification of the major circulating strains of NDV in wild birds and for calculating their genetic relatedness. Given the economic importance of the poultry industry, the present findings reveal the necessity of the application of more strict hygienic measures and management practices in the poultry industry to prevent contact between wild birds and poultry flocks in order to avoid possible spreading of the infection. Our data suggest future research to compare NDV from wild birds and poultry flocks. Obtaining this information would help better understand the epidemiological pattern and transmission dynamics and consequently combat this viral disease.

## Figures and Tables

**Table 1:** Details of collected samples, including chicken flock size, type of poultry farm, location of farms, collection date, and number of collected wild birds.

Chicken Farm No.	Chicken Flock Size	Type of Poultry Farms	Farm Location	Collection Date (Month/Year)	Number of Collected Wild Birds
1	3000	Broiler	El-Hamoul	10/2017	Five cattle egrets and five house sparrows were collected from the vicinity of each farm.
2	2000	Broiler	El-Hamoul	12/2017	
3	4000	Layer	Kafrelsheikh	3/2018	
4	2500	Broiler	Balteem	5/2018	
5	5000	Broiler	Motobas	6/2018	
6	3200	Layer	El-Hamoul	11/2018	
7	4500	Broiler	Kafrelsheikh	1/2019	
8	5000	Broiler	Motobas	3/2019	
9	2700	Broiler	Kafrelsheikh	5/2019	
10	5000	Layer	Balteem	10/2019	

**Table 2:** Details of the two sets of primers and probes used for amplification of the NDV gene fragment in real-time reverse transcriptase polymerase chain reaction (RRT-PCR) and RT-PCR.

Primer	Sequence (5’–3’) Direction	Product Size (bp)	Reference
F+4839	TCCGGAGGATACAAGGGTCT	101	[41]
F+4894 (VFP-1)	[FAM]AAGCGTTTCTGTCTCCTTCCTCCA[TAMRA]		
F-4939	AGCTGTTGCAACCCCAAG		
NDV-F330 forward	AGGAAGGAGACAAAAACGTTTTATAGG	370	[42]
NDV-R700 reverse	TCAGCTGAGTTAATGCAGGGGAGG		

**Table 3:** Details of collected samples, including the number of collected wild birds; clinical signs; postmortem lesions; and results of RRT-PCR, RT-PCR, and sequencing.

Farm No.	Wild Bird No.	Clinical Signs	Post-mortem Changes	RRT-PCR (Positive/Total)	RT-PCR (Positive/Total)	Sequencing
1	5 cattle egrets	Whitish-green diarrhea (1/5)	Enteritis (1/5)	3/5	2/5	KFS-Elhamoul-1 strain
	5 house sparrows	No signs	No PM lesions	2/5	2/5	
2	5 cattle egrets	No signs	No PM lesions	1/5	1/5	
	5 house sparrows	Whitish-green diarrhea (1/5)	Enteritis (1/5)	2/5	1/5	KFS-Elhamoul-3 strain
3	5 cattle egrets	Ruffled feathers (1/5)	Air sac cloudiness (1/5)	3/5	3/5	
	5 house sparrows	No signs	No PM lesions	1/5	1/5	
4	5 cattle egrets	No signs	No PM lesions	2/5	1/5	
	5 house sparrows	Ruffled feathers (2/5)	No PM lesions	1/5	1/5	
5	5 cattle egrets	Whitish-green diarrhea (1/5)	Enteritis (1/5)	2/5	2/5	KFS-Motobas-2 strain
	5 house sparrows	No signs	No PM lesions	1/5	1/5	
6	5 cattle egrets	Ruffled feathers (1/5)	No PM lesions	2/5	2/5	
	5 house sparrows	No signs	No PM lesions	1/5	0/5	
7	5 cattle egrets	No signs	No PM lesions	3/5	2/5	
	5 house sparrows	Whitish-green diarrhea (1/5)	Enteritis (1/5)	2/5	1/5	
8	5 cattle egrets	Whitish-green diarrhea (1/5)	Enteritis (1/5)	3/5	3/5	
	5 house sparrows	No signs	No PM lesions	2/5	2/5	
9	5 cattle egrets	Ruffled feathers (2/5)	No PM lesions	2/5	2/5	
	5 house sparrows	Ruffled feathers	No PM lesions	1/5	1/5	
10	5 cattle egrets	No signs	No PM lesions	1/5	0/5	
	5 house sparrows	No signs	No PM lesions	0/5	0/5	

**Table 4:** Clinical signs appeared in susceptible chickens following pathogenicity testing of sub-genotype VII.1.1 NDV strains originating in wild birds.

Group	Clinical Signs	Number of Affected Birds (Days Post-Inoculation)								Total % Mortality
		3rd	4th	5th	6th	7th	8th	9th	10th	
	Depression and ruffled feathers	12/40	30/34	26/30	23/25	21/24	8/14	6/12	3/9	
	Decreased feed intake	12/40	25/34	26/30	23/25	21/24	8/14	6/12	3/9	
G1	Greenish diarrhea	0/40	15/34	25/30	21/25	20/24	7/14	5/12	2/9	
	Swollen head	0/40	17/34	23/30	20/25	20/24	7/14	5/12	2/9	
	Respiratory signs	0/40	17/34	23/30	20/25	20/24	7/14	5/12	2/9	
	Nervous signs	0/40	0/34	9/30	18/25	17/24	10/14	9/12	6/9	
	Mortality	3/40	4/34	5/30	1/25	7/24	2/14	3/12	3/9	32/40 (80%)
	Depression and ruffled feathers	10/40	30/34	28/31	25/27	19/23	13/17	10/14	7/11	
	Decreased feed intake	10/40	27/34	29/31	25/27	19/23	13/17	10/14	7/11	
G2	Greenish diarrhea	0/40	17/34	22/31	21/27	15/23	9/17	6/14	3/11	
	Swollen head	0/40	12/34	17/31	16/27	16/23	10/17	7/14	4/11	
	Respiratory signs	0/40	12/34	17/31	16/27	16/23	10/17	7/14	4/11	
	Nervous signs	0/40	0/34	8/31	19/27	16/23	12/17	9/14	6/11	
	Mortality	3/40	3/34	4/31	4/27	3/23	3/17	3/14	4/11	27/40 (67.5%)
	Depression and ruffled feathers	9/40	26/35	28/31	26/28	22/24	15/17	10/12	8/10	
G3	Decreased feed intake	9/40	29/35	29/31	25/28	22/24	15/17	10/12	8/10	
	Greenish diarrhea	0/40	17/35	21/31	22/28	18/24	11/17	7/12	5/10	
	Swollen head	0/40	16/35	19/31	19/28	15/24	9/17	5/12	3/10	
	Respiratory signs	0/40	16/35	19/31	19/28	15/24	9/17	5/12	3/10	
	Nervous signs	0/40	0/35	7/31	12/28	13/24	10/17	5/12	6/10	
	Mortality	2/40	4/35	3/31	4/28	4/24	5/17	2/12	1/10	25/40 (62.5%)
G4	Clinical signs	0/40	0/40	0/40	0/40	0/40	0/40	0/40	0/40	
	Mortality	0/40	0/40	0/40	0/40	0/40	0/40	0/40	0/40	0/40 (0%)

**Table 5:** Post-mortem changes reported in susceptible chickens following pathogenicity testing.

Group	Post-Mortem Changes			Lesions in Dead Birds (Days Post-Inoculation)							
				3rd	4th	5th	6th	7th	8th	9th	10th
	No. of examined birds		Dead	3	4	5	1	7	2	3	3
			Euthanized	3	0	0	0	3	0	0	3
	Hemorrhagic tracheitis			0/6	3/4	3/5	0/1	9/10	2/2	2/3	6/6
G1	Lung congestion			0/6	3/4	3/5	0/1	8/10	1/2	2/3	6/6
	Hemorrhagic cecal tonsils			1/6	2/4	3/5	0/1	6/10	1/2	3/3	5/6
	Enlarged spleen			1/6	4/4	5/5	1/1	9/10	2/2	3/3	5/6
	Hemorrhages on the periventricular gland tips			0/6	3/4	3/5	1/1	10/10	2/2	2/3	6/6
	Greenish proventricular and intestinal contents			0/6	3/4	5/5	1/1	8/10	2/2	3/3	6/6
	Hemorrhagic enteritis			0/6	3/4	3/5	1/1	6/10	1/2	2/3	5/6
	No. of examined birds	Dead		3	3	4	4	3	3	3	4
		Euthanized		3	0	0	0	3	0	0	3
G2	Hemorrhagic tracheitis			0/6	2/3	2/4	3/4	4/6	2/3	1/3	6/7
	Lung congestion			0/6	3/3	3/4	2/4	4/6	1/3	0/3	5/7
	Hemorrhagic cecal tonsils			2/6	2/3	1/4	3/4	3/6	1/3	2/3	3/7
	Enlarged spleen			4/6	3/3	4/4	4/4	6/6	3/3	3/3	6/7
	Hemorrhages on the periventricular gland tips			0/6	3/3	4/4	3/4	3/6	2/3	1/3	7/7
	Greenish proventricular			0/6	3/3	4/4	4/4	4/6	3/3	3/3	7/7
	Hemorrhagic enteritis			0/6	2/3	2/4	4/4	3/6	1/3	1/3	5/7
	No. of examined birds		Dead	2	4	3	4	4	5	2	1
			Euthanized	3	0	0	0	3	0	0	3
G3	Hemorrhagic tracheitis			0/5	3/4	1/3	1/4	5/7	2/5	0/2	3/4
	Lung congestion			0/5	2/4	2/3	2/4	6/7	3/5	1/2	2/4
	Hemorrhagic cecal tonsils			0/5	3/4	2/3	3/4	5/7	4/5	0/2	0/4
	Enlarged spleen			2/5	4/4	3/3	3/4	7/7	4/5	1/2	4/4
	Hemorrhages on the periventricular gland tips			0/5	2/4	3/3	2/4	7/7	2/5	2/2	3/4
	Greenish proventricular and intestinal contents			0/5	3/4	3/3	4/4	7/7	5/5	2/2	4/4
	Hemorrhagic enteritis			0/5	1/4	2/3	1/4	5/7	3/5	0/2	1/4
G4	No. of examined birds		Dead	0	0	0	0	0	0	0	0
			Euthanized	3	0	0	0	3	0	0	3
	PM lesions			0/3	0/0	0/0	0/0	0/3	0/0	0/0	0/3

## Supplementary Materials

The following are available online at https://www.mdpi.com/2076-2615/11/2/505/s1, Table S1: Diversity percent between studied NDV strains and other NDV strains from GenBank using MEGA X software.

## References

[1] Alexander D.J., Aldous E.W., Fuller C.M. (2012). The long view: A selective review of 40 years of Newcastle disease research. Avian Pathol..

[2] OIE World Animal Health Information Database (WAHIS Interface)—Version 1. http://www.oie.int/wahis_2/public/wahid.php/Wahidhome/Home.

[3] (2011). World Livestock Disease Atlas: A Quantitative Analysis of GlobalAnimal Health Data A (2006–2009).

[4] Miller P.J., Koch G. (2013). Newcastle disease. Dis. Poult..

[5] Bello M.B., Yusoff K.M., Ideris A., Hair-Bejo M., Peeters B.P.H., Jibril A.H., Tambuwal F.M., Omar A.R. (2018). Genotype Diversity of Newcastle Disease Virus in Nigeria: Disease Control Challenges and Future Outlook. Adv. Virol..

[6] Absalon A.E., Cortes-Espinosa D.V., Lucio E., Miller P.J., Afonso C.L. (2019). Epidemiology, control, and prevention of Newcastle disease in endemic regions: Latin America. Trop. Anim. Health Prod..

[7] Kapczynski D.R., Afonso C.L., Miller P.J. (2013). Immune responses of poultry to Newcastle disease virus. Dev. Comp. Immunol..

[8] OIE (2012). Newcastle disease. OIE Manual of Standards for Diagnostic Tests and Vaccines.

[9] ICTV (2019). International Committee on Taxonomy of Viruses Virus Taxonomy: 2018b Release. https://talk.ictvonline.org/taxonomy/.

[10] Moura V., Susta L., Cardenas-Garcia S., Stanton J., Miller P., Afonso C., Brown C. (2016). Neuropathogenic capacity of lentogenic, mesogenic, and velogenic Newcastle disease virus strains in day-old chickens. Vet. Pathol..

[11] Dimitrov K.M., Abolnik C., Afonso C.L., Albina E., Bahl J., Berg M., Briand F.-X., Brown I.H., Choi K.-S., Chvala I. (2019). Updated unified phylogenetic classification system and revised nomenclature for Newcastle disease virus. Infect. Genet. Evol..

[12] Xue C., Cong Y., Yin R., Sun Y., Ding C., Yu S., Liu X., Hu S., Qian J., Yuan Q. (2017). Genetic diversity of the genotype VII Newcastle disease virus: Identification of a novel VIIj sub-genotype. Virus genes.

[13] Dimitrov K., Dong-Hun L., Williams-Coplin D., Olivier T., Miller P., Afonso C. (2016). Newcastle Disease Viruses Causing Recent Outbreaks Worldwide Show Unexpectedly High Genetic Similarity with Historical Virulent Isolates from the 1940’s. J. Clin. Microbiol..

[14] Snoeck C., Owoade A., Couacy-Hymann E., Alkali B., Okwen M., Adeyanju T., Komoyo G.F., Nakouné E., Faou A., Muller C. (2013). High Genetic Diversity of Newcastle Disease Virus in Poultry in West and Central Africa: Cocirculation of Genotype XIV and Newly Defined Genotypes XVII and XVIII. J. Clin. Microbiol..

[15] Diel D.G., da Silva L.H., Liu H., Wang Z., Miller P.J., Afonso C.L. (2012). Genetic diversity of avian paramyxovirus type 1: Proposal for a unified nomenclature and classification system of Newcastle disease virus genotypes. Infect. Genet. Evol..

[16] Abolnik C., Mubamba C., Wandrag D.B., Horner R., Gummow B., Dautu G., Bisschop S.P. (2018). Tracing the origins of genotype VII h Newcastle disease in Southern Africa. Transbound. Emerg. Dis..

[17] Fuller C., Löndt B., Dimitrov K., Lewis N., van Boheemen S., Fouchier R., Coven F., Goujgoulova G., Haddas R., Brown I. (2017). An epizootiological report of the re-emergence and spread of a lineage of virulent Newcastle disease virus into Eastern Europe. Transbound. Emerg. Dis..

[18] Kammon A., Monne I., Asheg A., Cattoli G. (2018). Molecular detection and characterisation of avian paramyxovirus type 1 in backyard chickens and pigeons in Alzintan city of Libya. Open Vet. J..

[19] Mapaco L.P., Monjane I.V., Nhamusso A.E., Viljoen G.J., Dundon W.G., Achá S.J. (2016). Phylogenetic analysis of Newcastle disease viruses isolated from commercial poultry in Mozambique (2011–2016). Virus Genes.

[20] Jindal N., Chander Y., Chockalingam A.K., De Abin M., Redig P.T., Goyal S.M. (2009). Phylogenetic analysis of Newcastle disease viruses isolated from waterfowl in the upper midwest region of the United States. Virol. J..

[21] Miller P.J., Haddas R., Simanov L., Lublin A., Rehmani S.F., Wajid A., Bibi T., Khan T.A., Yaqub T., Setiyaningsih S. (2015). Identification of new sub-genotypes of virulent Newcastle disease virus with potential panzootic features. Infect. Genet. Evol..

[22] Molini U., Aikukutu G., Khaiseb S., Cattoli G., Dundon W.G. (2017). First genetic characterization of Newcastle disease viruses from Namibia: Identification of a novel VIIk subgenotype. Arch. Virol..

[23] Radwan M.M., Darwish S.F., El-Sabagh I.M., El-Sanousi A.A., Shalaby M.A. (2013). Isolation and molecular characterization of Newcastle disease virus genotypes II and VIId in Egypt between 2011 and 2012. Virus Genes.

[24] Brown V.R., Bevins S.N. (2017). A review of virulent Newcastle disease viruses in the United States and the role of wild birds in viral persistence and spread. Vet. Res..

[25] Sabban M., Zied A.A., Basyouni A., Nadiem S., Barhouma N., Habashi Y. (1982). Susceptibility and possible role of doves in transmission of Newcastle disease in Egypt. Zent. Vet. Reihe B.

[26] Mayo M.A. (2002). Virus taxonomy—Houston 2002. Arch. Virol..

[27] Perttula L. (2010). Epidemiology and Characterization of Newcastle Disease in Smallholder Poultry in Mozambique.

[28] Aziz-ul-Rahman M.H., Shabbir M.Z. (2018). Suppl-2, M3: Adaptation of Newcastle Disease Virus (NDV) in Feral Birds and their Potential Role in Interspecies Transmission. Open Virol. J..

[29] Seal B., Wise M., Pedersen J., Senne D., Alvarez R., Scott M., King D., Yu Q., Kapczynski D. (2005). Genomic sequences of low-virulence avian paramyxovirus-1 (Newcastle disease virus) isolates obtained from live-bird markets in North America not related to commonly utilized commercial vaccine strains. Vet. Microbiol..

[30] Jørgensen H.P., Handberg K., Ahrens P., Therkildsen O., Manvell R., Alexander D. (2004). Strains of avian paramyxovirus type 1 of low pathogenicity for chickens isolated from poultry and wild birds in Denmark. Vet. Record.

[31] Dimitrov K., Ramey A., Qiu X., Bahl J., Afonso C. (2016). Temporal, geographic, and host distribution of avian paramyxovirus 1 (Newcastle disease virus). Infect. Genet. Evol..

[32] Mosad S.M., El-Gohary F.A., Ali H.S., El-Sharkawy H., Elmahallawy E.K. (2020). Pathological and Molecular Characterization of H5 Avian Influenza Virus in Poultry Flocks from Egypt over a Ten-Year Period (2009–2019). Animals (Basel).

[33] Ramey A.M., Goraichuk I.V., Hicks J.T., Dimitrov K.M., Poulson R.L., Stallknecht D.E., Bahl J., Afonso C.L. (2017). Assessment of contemporary genetic diversity and inter-taxa/inter-region exchange of avian paramyxovirus serotype 1 in wild birds sampled in North America. Virol. J..

[34] Fagbohun O., Oluwayelu D., Owoade A., Olayemi F. (2000). Survey for antibodies to Newcastle disease virus in cattle egrets, pigeons and Nigerians laughing doves. Afr. J. Biomed. Res..

[35] Hicks J.T., Dimitrov K.M., Afonso C.L., Ramey A.M., Bahl J. (2019). Global phylodynamic analysis of avian paramyxovirus-1 provides evidence of inter-host transmission and intercontinental spatial diffusion. BMC Evol. Biol..

[36] Turan N., Ozsemir C., Yilmaz A., Cizmecigil U.Y., Aydin O., Bamac O.E., Gurel A., Kutukcu A., Ozsemir K., Tali H.E. (2020). Identification of Newcastle disease virus subgenotype VII.2 in wild birds in Turkey. BMC Vet. Res..

[37] Maiti S.K., Tiwary R., Vasan P., Dutta A. (2006). Xylazine, diazepam and midazolam premedicated ketamine anaesthesia in white Leghorn cockerels for typhlectomy. J. S. Afr. Vet. Assoc..

[38] Grimes S.E. (2002). A Basic Laboratory Manual for the Small-Scale Production and Testing of I-2 Newcastle Disease Vaccine.

[39] Awad M., Mosad S., El-Kenawy A. (2020). Molecular differentiation between velogenic isolates and lentogenic LaSota strain of Newcastle disease virus. Mansoura Vet. Med. J..

[40] Wise M.G., Suarez D.L., Seal B.S., Pedersen J.C., Senne D.A., King D.J., Kapczynski D.R., Spackman E. (2004). Development of a real-time reverse-transcription PCR for detection of newcastle disease virus RNA in clinical samples. J. Clin. Microbiol..

[41] Selim K.M., Selim A., Arafa A., Hussein H.A., Elsanousi A.A. (2018). Molecular characterization of full fusion protein (F) of Newcastle disease virus genotype VIId isolated from Egypt during 2012-2016. Vet. World.

[42] Kumar S., Stecher G., Li M., Knyaz C., Tamura K. (2018). MEGA X: Molecular Evolutionary Genetics Analysis across Computing Platforms. Mol. Biol. Evol..

[43] Hall T. (1999). BioEdit: A user-friendly biological sequence alignment editor and analysis program for Windows 95/98/NT. Nucleic Acids Symposium.

[44] Ali H.H. (2015). Study serologic status of newcastle disease in broilers chikens by haemagglutination inhibition test in Suliamania province. Glob. J. Bio-Sci. Biotechnol..

[45] Reed L.J., Muench H. (1938). A simple method of estimating fifty per cent endpoints. Am. J. Epidemiol..

[46] Ahmed A., Kandeil A., Kutkat M., Abdel-Moez A. (2019). Advancement in Vaccination of Broiler Chickens with Genotype-Matched Vaccines to Currently Epidemic Newcastle Disease Virus Genotype VII in Egypt. J. World’s Poult. Res..

[47] Downie T. (1990). Theory and practice of histological techniques edited by JD Bancroft & a. Stevens, Churchill Livingstone, Edinburgh, 740 pages, £ 55.00. Histopathology.

[48] Abdisa T., Tagesu T. (2017). Review on Newcastle Disease in Poultry and its Public Health Importance. J. Anim. Poult. Sci..

[49] Silva J., Mota R., Vilela S., Doretto Júnior L., Pinheiro Júnior J., Silva L. (2006). Newcastle disease virus infection in Sparrows (Passer domesticus, Linneaus, 1758) captured in poultry farms of the Agreste region of the State of Pernambuco. Braz. J. Poult. Sci..

[50] Munir T., Aslam A., Zahid B., Ahmed I., Imran M., Ijaz M. (2015). Potential of commonly resident wild birds towards newcastle disease virus transmission. Pak. Vet. J..

[51] Lindh E., Ek-Kommonen C., Vaananen V.M., Alasaari J., Vaheri A., Vapalahti O., Huovilainen A. (2012). Molecular epidemiology of outbreak-associated and wild-waterfowl-derived newcastle disease virus strains in Finland, including a novel class I genotype. J. Clin. Microbiol..

[52] Aldous E.W., Mynn J.K., Banks J., Alexander D.J. (2003). A molecular epidemiological study of avian paramyxovirus type 1 (Newcastle disease virus) isolates by phylogenetic analysis of a partial nucleotide sequence of the fusion protein gene. Avian Pathol..

[53] Blaxland J. (1951). Newcastle disease in shags and cormorants and its significance as a factor in the spread of this disease among domestic poultry. Vet. Rec..

[54] Dhama K., Mahendran M., Tomar S. (2008). Pathogens Transmitted by Migratory Birds: Threat Perceptions to Poultry Health and Production. Int. J. Poult. Sci..

[55] Rehman Z.U., Meng C., Sun Y., Mahrose K.M., Umar S., Ding C., Munir M. (2018). Pathobiology of Avian avulavirus 1: Special focus on waterfowl. Vet. Res..

[56] Elmberg J., Berg C., Lerner H., Waldenström J., Hessel R. (2017). Potential disease transmission from wild geese and swans to livestock, poultry and humans: A review of the scientific literature from a One Health perspective. Infect. Ecol. Epidemiol..

[57] Saif B.G.J., Fadly L.R.M., Swayne D. (2005). Diseases of Poultry.

[58] Alexander D.J. (2009). Ecology and epidemiology of Newcastle disease. Avian Influenza and Newcastle Disease.

[59] Alexander D. (2000). Newcastle disease and other avian paramyxoviruses. Rev. Sci. Tech. Off. Int. Epizoo..

[60] Alexander D., Banks J., Collins M., Manvell R., Frost K., Speidel E., Aldous E. (1999). Antigenic and genetic characerisation of Newcastle disease viruses isolated from outbreaks in domestic fowl and turkeys in Great Britain during 1997. Vet. Rec..

[61] Guirguis W.I. (1983). The Role Played by white Egrets in Transmission of Newcastle Disease Virus (Velogenicviscerotropic Strains). Master’s Thesis.

[62] Xu H., Lohr J., Greiner M. (1997). The selection of ELISA cut-off points for testing antibody to Newcastle disease by two-graph receiver operating characteristic (TG-ROC) analysis. J. Immunol. Methods.

[63] Zhang L., Liu N., Ma X., Jiang L. (2013). The transcriptional control machinery as well as the cell wall integrity and its regulation are involved in the detoxification of the organic solvent dimethyl sulfoxide in Saccharomyces cerevisiae. FEMS Yeast Res..

[64] Alexander D., Gough R., Saif Y., Barnes H., Glisson J., Fadly A., McDougald L., Swayne D. (2003). Newcastle Disease, Other Avian Paramyxoviruses, and Pneumovirus Infections. Disease of Poultry.

[65] Mazumder A., Khatun S., Nooruzzaman M., Chowdhury E., Das P., Islam M. (2012). Isolation and identification of Newcastle disease viruses from field outbreaks in chickens and pigeons. Bangladesh Vet..

[66] Williams R., Boshoff C.H., Verwoerd D., Schoeman M., van Wyk A., Gerdes T.H., Roos K. (1997). Detection of antibodies to Newcastle disease virus in ostriches (Struthio camelus) by an indirect ELISA. Avian Dis..

[67] Suarez D.L., Miller P.J., Koch G., Mundt E., Rautenschlein S. (2020). Newcastle disease, other avian paramyxoviruses, and avian metapneumovirus infections. Dis. Poult..

[68] Aldous E., Alexander D. (2001). Detection and differentiation of Newcastle disease virus (avian paramyxovirus type 1). Avian Pathol..

[69] Beguas R., Umali D. (2018). Genetic characterization of Newcastle Disease virus from broiler flocks in selected areas in Central Luzon, Philippines. Philipp. J. Vet. Med..

[70] El Naggar R.F., Rohaim M.A., Bazid A.H., Ahmed K.A., Hussein H.A., Munir M. (2018). Biological characterization of wild-bird-origin avian avulavirus 1 and efficacy of currently applied vaccines against potential infection in commercial poultry. Arch. Virol..

[71] Schelling E., Thur B., Griot C., Audige L. (1999). Epidemiological study of Newcastle disease in backyard poultry and wild bird populations in Switzerland. Avian Pathol..

[72] Xu X., Renfu Y., Qian J., Sun Y., Wang C., Ding C., Yu S., Hu Z., Liu X., Cong Y. (2017). Identification and pathotypical analysis of a novel VIk sub-genotype Newcastle disease virus obtained from pigeon in China. Virus Res..

[73] Kim L.M., King D.J., Curry P.E., Suarez D.L., Swayne D.E., Stallknecht D.E., Slemons R.D., Pedersen J.C., Senne D.A., Winker K. (2007). Phylogenetic diversity among low-virulence newcastle disease viruses from waterfowl and shorebirds and comparison of genotype distributions to those of poultry-origin isolates. J. Virol..

[74] Thomazelli L., de Araujo J., Ferreira C.d.S., Hurtado R., Oliveira D., Ometto T., Golono M., Sanfilippo L., Demetrio C., Figueiredo M. (2012). Molecular surveillance of the Newcastle disease virus in domestic and wild birds on the North Eastern Coast and Amazon biome of Brazil. Braz. J. Poult. Sci..

[75] Nunes C.F., Fonseca F., Leite A.T.M., Silva Filho R.P.d., Finger P.F., Castro C.C., Fischer G., Vargas G., Hübner S.d.O. (2012). Investigation on Newcastle disease virus (NDV), infectious bursal disease virus (IBDV) and avian poxvirus (APV) in Magellanic Penguins in Southern Region of Brazil. Braz. Arch. Biol. Technol..

[76] Daodu O.B., Aiyedun J.O., Kadir R.A., Ambali H.M., Oludairo O.O., Olorunshola I.D., Daodu O.C., Baba S.S. (2019). Awareness and antibody detection of Newcastle disease virus in a neglected society in Nigeria. Vet. World.

[77] van Seventer J.M., Hochberg N.S. (2017). Principles of Infectious Diseases: Transmission, Diagnosis, Prevention, and Control. Int. Encycl. Public Health.

[78] Mariappan A.K., Munusamy P., Kumar D., Latheef S.K., Singh S.D., Singh R., Dhama K. (2018). Pathological and molecular investigation of velogenic viscerotropic Newcastle disease outbreak in a vaccinated chicken flocks. Virusdisease.

[79] Bhaiyat M., Ochiai K., Itakura C., Islam M., Kida H. (1994). Brain lesions in young broiler chickens naturally infected with a mesogenic strain of Newcastle disease virus. Avian Pathol..

